# Equal Distress and Less Hope in Parkinson's Disease Patients Compared to Brain Tumors Patients

**DOI:** 10.1002/mdc3.14355

**Published:** 2025-02-12

**Authors:** Simone D'Souza, Esther Tekampe, Marco Skardelly, Stephan Zipfel, Martin Teufel, Björn Falkenburger

**Affiliations:** ^1^ Department of Neurology University Hospital Carl Gustav Dresden Dresden Germany; ^2^ Department of Psychosomatic Medicine and Psychotherapy University Hospital Tübingen Tübingen Germany; ^3^ Center for Neuro‐Oncology, Comprehensive Cancer Center Tübingen‐Stuttgart University Hospital Tübingen Tübingen Germany; ^4^ Department of Neurosurgery University Hospital Tübingen Tübingen Germany; ^5^ German Centre of Mental Health (DZPG) University of Tübingen Tübingen Germany; ^6^ Section of Psycho‐Oncology, West German Cancer Center (WTZ) University Hospital Duisburg‐Essen Essen Germany; ^7^ Department of Psychosomatic Medicine and Psychotherapy LVR University Hospital Duisburg‐Essen Essen Germany; ^8^ Centre for Translational Neuro‐ and Behavioral Sciences (C‐TNBS) University of Duisburg‐Essen Essen Germany

**Keywords:** Parkinson's disease, brain tumor, psychosocial counseling, distress, depression, anxiety

This study examined the psychosocial needs of patients with Parkinson's disease (PwPD) in comparison to patients with brain tumors (PwBT), given the absence of any comparable psychosocial support for PwPD, despite the high demand for psychosocial care.^1^ On the basis of two previously characterized cohorts, statistical analyses were conducted utilizing the Patient Health Questionnaire‐2 (PHQ‐2), the Generalized Anxiety Disorder‐2 (GAD‐2), the Distress Thermometer (DT) and the Herth Hope Index (HHI). For method details, see Supplemental File S1. The univariate analysis results are in Table 1. Multivariate analysis showed a significant difference between PwPD and PwBT in the sum scores of the PHQ‐2, GAD‐2, DT, and HHI (one‐way MANOVA, *F*(4, 161) = 19.39, *P* < 0.001, partial η^2^ = 0.325, Wilk's Λ = 0.675). Post‐hoc analyses (univariate ANOVAs) indicate that PwBT are more likely to be depressed (PHQ‐2, *F*(1, 164) = 4.764, *P* = 0.030, partial η^2^ = 0.028) and anxious (GAD‐2, *F*(1, 164) = 4.546 *P* < 0.001, partial η^2^ = 0.027), whereas PwPD show comparably high distress levels (DT, *F*(1, 166) = 0.033, *P* = 0.856, partial η^2^ = 0.000) and experience less hope (HHI, *F*(1, 164) = 36.873, *P* < 0.001, partial η^2^ = 0.184). The ANOVA effect size of the HHI total score was large (η^2^ = 0.184), whereas the effect sizes for the PHQ‐2 and GAD‐2 total scores were small (η^2^ = 0.028 and η^2^ = 0.027, respectively). Showing similar distress and less hope levels, these findings indicate that psychosocial screening should be provided to PwPD to the same extent as to PwBT. The Distress Thermometer could be an effective tool for this purpose.^2^, ^3^ Moreover, PwPD showed a medium correlation between the HHI and PHQ‐2 scores (*r* = −0.496; *P* < 0.001), GAD‐2 score (*r* = −0.317; *P* = 0.001), and DT score (*r* = −0.315; *P* = 0.001), as well as a small correlation with physical problems (*r* = −0.244; *P* = 0.014) and emotional problems (*r* = −0.213; *P* = 0.031). PwBT showed a strong correlation between the HHI and PHQ‐2 scores (*r* = −0.496; *P* < 0.001), and a medium correlation with the GAD‐2 score (*r* = −0.496; *P* < 0.001) and DT score (*r* = −0.496; *P* < 0.001). Hope negatively correlated with anxiety, depression, and distress in both groups. In PwPD, hope also weakly correlated with physical and emotional problems, suggesting that motor and non‐motor symptoms may reduce hope. Conversely, effective treatment for both motor and non‐motor symptoms could improve hope. One limitation of this study is that the majority of patients in both groups had mild disease severity (PwPD H & Y stages I/II = 56.2%; PwBT WHO stages I/II = 65.2%). A study population with more severely affected patients may yield different results on anxiety and depression. For limitations details, see Supplemental File S2. In conclusion, these findings strongly recommend integrating an structured screening and clinical treatment pathway for psychosocial problems into routine clinical practice for PwPD, mirroring the approach used for PwBT.^4^, ^5^

**TABLE 1:** Demographic, clinical and psychosocial data

Feature	PwBT N = 66 (100%)	PwPD N = 105 (100%)	*P*‐value
Sex, female	38 (57.6)	37 (35.2)	**<0.001** ^C^
Age (years)	M 53.0 ± 11.1	M 65.7 ± 9.59	**<0.001** ^T^
With partner	51 (77.3)	82 (78.1)	**<0.001** ^C^
Single	6 (9.1)	21 (20.0)	
Others	9 (13.6)	0 (0.0)	
Missing	0 (0.0)	2 (1.9)	
Alone	9 (13.6)	11 (10.5)	0.87^C^
With family	54 (81.8)	84 (80.0)	
Others	3 (4.5)	5 (4.8)	
Missing	0 (0.0)	5 (4.8)	
School (≥10 years)	7 (10.6)	19 (18.1)	**<0.001** ^C^
School (<10 years)	42 (63.6)	45 (42.9)	
University	14 (21.2)	36 (34.3)	
Others	3 (4.5)	0 (0.0)	
Missing	0 (0.0)	5 (4.8)	
Working	47 (71.2)	27 (25.7)	**<0.001** ^F^
Not working	19 (28.8)	77 (73.3)	
Missing	0 (0.0)	1 (1.0)	
WHO stage			
I	30 (45.5)		
II	13 (19.7)		
III	5 (7.6)		
IV	18 (27.3)		
Hoehn & Yahr stage			
I		8 (7.6)	
II		51 (48.6)	
III		38 (36.2)	
IV		5 (4.8)	
Missing		3 (2.9)	
Disease duration [years]	M 1.74 ± 3.85 min 0; max 20 IQR 0–2	M 8.91 ± 16.6 min 0; max 26 IQR 5–13	**<0.001** ^**U**^
*PHQ – 2 ≥ 3*	N = 14 (21.2)	12 (11.4)	0.098^C^
Missing	0 (0.0)	3 (2.9)	
*GAD – 2 ≥ 3*	N = 19 (28.8)	18 (17.1)	0.089^C^
Missing	0 (0.0)	3 (2.9)	
*HHI*	62 (93.9) M 42.4 ± 4.78 min 24; max 48 IQR 36–40	103 (98.1) M 36.83 ± 5.52 min 20; max 48 IQR 34–41	**<0.001** ^**U**^
HHI Missing	0 (0)	2 (1.9)	
*DT*	66 (100) M 5.26 ± 2.44 min 1; max 10 IQR 3–7	100 (95.2) M 5.19 ± 2.29 min 0; max 10 IQR 3–7	0.86^T^
Missing	0 (0.0)	5 (4.8)	
DT ≥4	41 (62.1)	66 (62.9)	0.86^F^
Problem list			
Physical problems	50 (75.8)	98 (93.3)	**<0.001** ^**F**^
Family problems	7 (10.6)	24 (22.9)	**0.04** ^**F**^
Emotional problems	52 (78.8)	69 (65.7)	0.16^F^
Spiritual problems	3 (4.5)	3 (2.9)	0.68^F^
Practical problems	12 (18.2)	31 (30.4)	0.10^F^
Coping parameters			
Expectations of therapeutic success	66 (100) M 2.22 ± 1.94 min 1; max 10 IQR 1–3	103 (98.1) M 4.91 ± 2.12 min 1; max 10 IQR 3–6	**<0.001** ^**U**^
Missing	0 (0.0)	2 (1.9)	
Expectations of side effects	66 (100) M 2.83 ± 1.91 min 1; max 10 IQR 1.375–4	100 (95.2) M 4.95 ± 2.18 min 1; max 10 IQR 3–7	**<0.001** ^**U**^
Missing	0 (0.0)	5 (4.8)	
Former resilience rating	54 (98.1) M 2.93 ± 2.12 min 1; max 9 IQR 1–4	100 (90.1) M 3.11 ± 1.93 min 1; max 10 IQR 2–4	0.351^U^
Missing	12 (18.2)	5 (4.8)	
Current state of mental health	66 (100) M 4.15 ± 1.85 min 1; max 9 IQR 2–5.625	101 (96.2) M 5.48 ± 2.38 min 1; max 10 IQR 4–8	**0.001** ^**U**^
Missing	0 (0)	4 (3.8)	
Ability to handle the disease	66 (100) M 3.18 ± 1.85 min 1; max 9 IQR 2–5	102 (97.1) M 4.27 ± 2.21 min 1; max 10 IQR 2.75–6	**0.001** ^**U**^
Missing	0 (0.0)	3 (2.9)	
Social environment support	66 (100) M 1.61 ± 1.35 min 1; max 10 IQR 1–2	103 (98.1) M 2.68 ± 1.66 min 1; max 8 IQR 1–4	**<0.001** ^**U**^
Missing	0 (0.0)	2 (1.9)	
Psychotherapeutic treatment			
Never	48 (72.7)	71 (67.6)	0.21^C^
Current	4 (6.1)	15 (14.3)	
Former	14 (21.2)	17 (16.2)	
Missing		2 (1.9)	
Psychotropic medication			
Never	55 (83.3)	74 (70.5)	0.184 ^C^
Every day	8 (12.1)	24 (22.9)	
Occasional	3 (4.5)	4 (3.8)	
Missing	0 (0.0)	3 (2.9)	

Bold values indicates a significant value *p* < 0.05.

Abbreviations: WHO, World Health Organization; PHQ, Patient Health Questionnaire; GAD, Generalized Anxiety Disorder; HHI, Herth‐Hope‐Index; DT, Distress Thermometer, BT, Brain Tumor; PD, Parkinson's disease; M, Mean; min, minimum; max, maximum; IQR, interquartile range; U, Mann–Whitney U test; C, Chi‐square test; T, T test; F, Fisher's exact test.

## Author Roles

(1) Research project: A. Conception, B. Organization, C. Execution; (2) Statistical Analysis: A. Design, B. Execution, C. Review and Critique; (3) Manuscript Preparation: A. Writing of the first draft, B. Review and Critique;

S.D.S.: 1A, 1B, 1C, 2A, 2B, 2C, 3A, 3B

E.T.: 1B, 1C, 2B

M.S.: 1A, 1B, 1C

S.Z.: 1B

M.T.: 1A, 1B, 3B

B.F.: 1A, 2B, 3B

Editing and approval of manuscript: All authors.

## Disclosures

**Ethical Compliance Statement:** All patients gave written informed consent to participate in this study. The study was approved by the ethics committee at Technische Universität Dresden (IRB00001473, EK 37012019) and by the ethics committee at University Hospital Tübingen (reference number 602/2014BO2) and conducted in accordance with relevant guidelines and regulations. We confirm that all authors have read the Journal's position on issues involved in ethical publication and affirm that this work is consistent with those guidelines.

**Funding Sources and Conflict of Interest:** The authors declare that there are no funding sources or conflicts of interest relevant to this work.

**Financial Disclosures for the previous 12 months:** The authors declare that there are no additional disclosures to report.

## Supporting information


**File S1.** Methods.

**File S2.** Limitations.

## Data Availability

The data that support the findings of this study are available from the corresponding author upon reasonable request.
