# Supplementary material for: Equal Distress and Less Hope in Parkinson's Disease Patients Compared to Brain Tumors Patients
Source: Mov Disord Clin Pract. 2025 Feb 12;12(5):709–12. doi: 10.1002/mdc3.14355 (PMC12070159; doi:10.1002/mdc3.14355)
Supplement: Supplementary file 1 — File S1. Methods. [file MDC3-12-709-s001.docx]

**Additional limitations of the manuscript “Equal distress and less hope in Parkinson's disease patients compared to brain tumors patients**”

(1) We conducted a post-hoc analysis of two medical centers. A prospective, multi-center study will contribute to a better generalizability of the conclusions. (2) It is not possible to draw any predictive conclusions due to the cross-sectional study design. In order to draw conclusive and valid conclusions, longitudinal studies with a larger sample size are required. (3) The varying degrees of ability of PwPD and PwBT to influence the course of their respective illnesses (e.g., through physical and cognitive activity, regular medication, and good nutrition in Parkinson's disease) may complicate comparisons of hope and the need for psychosocial support in these two conditions. Recognizing Parkinson's disease as a manageable condition over a long period of time may have a positive impact on the perception of hope and psychosocial distress. However, additional work addressing this topic may be needed. (4) While Parkinson's disease progresses gradually and inevitably, the progression type differs significantly for diseases such as meningiomas and glioblastomas, an aspect that may impact the patient's psychosocial burden. However, stratifying patients by tumor grade and Hoehn-Yahr stage may narrow this knowledge gap. Nevertheless, early psychosocial intervention benefits both PwPD and PwBT.
