# Supplementary material for: Equal Distress and Less Hope in Parkinson's Disease Patients Compared to Brain Tumors Patients
Source: Mov Disord Clin Pract. 2025 Feb 12;12(5):709–12. doi: 10.1002/mdc3.14355 (PMC12070159; doi:10.1002/mdc3.14355)
Supplement: Supplementary file 2 — File S2. Limitations. [file MDC3-12-709-s002.docx]

**Methods section of the manuscript “Equal distress and less hope in Parkinson's disease patients compared to brain tumors patients”**

In this study, we analyzed the following questionnaires additionally to demographic, clinical, and psychosocial data (Table 1) in two patient populations previously described in cross-sectional observational studies (DOI: <https://doi.org/10.3389/fpsyg.2021.642345> and DOI: <https://doi.org/10.1002/mdc3.13937>): Patient Health Questionnaire-2 (PHQ-2), Generalized Anxiety Disorder-2 (GAD-2), Herth Hope Index (HHI), Distress Thermometer (DT), and a self-developed Questionnaire for Coping Parameters. PwPD with Hoehn & Yahr Stage I – IV were consecutively recruited between January 2019 and October 2020 at the outpatient clinic of the Department of Neurology, University Hospital “Carl Gustav Carus” in Dresden (IRB00001473, EK 37012019). PwBT with WHO stage I – IV were consecutively recruited between September 2015 and November 2016 at the outpatient clinic of the Department of Neurology, University Hospital Tübingen (reference number 602/2014BO2). The results of questionnaires GAD-2, PHQ-2, and Coping Parameters questionnaire have not yet been published in relation to PD in the present study cohort. The study was conducted in accordance with all relevant guidelines and regulations. Inclusion criteria in both cohorts were: (1) sufficient cognitive ability to complete the questionnaires as determined by the treating physician; (2) proficiency of written and spoken German; (3) no schizophrenia or florid delusional disorder unrelated to the neurological disease.

Statistical analysis was performed using statistical program for social sciences (SPSS) version 27 (IBM New York). Differences between the two patient groups (PwPD vs. PwBT), were analyzed by means of Chi-square test for categorical variables, Mann-Whitney U test for metric scaled non-normally distributed variables, T-test for metric-scaled normally distributed variables, and Fisher’s exact test for small sample sizes (Tables 1). In addition, the coping parameter differences between the two patient groups were investigated by means of the Mann-Whitney U test. A multivariate analysis of variance (MANOVA) was conducted to identify differences of the two patient groups and the variables HHI, GAD-2, PHQ-2, and DT. Effect size was calculated using partial eta. According to Cohen, the limits for effect size are .01 (small effect), .06 (medium effect), and .14 (large effect). For correlation analysis, according to Cohen's r, *r* ≥ 0.1 was considered a small effect size, *r* ≥ 0.3 was considered a medium effect size, and *r* ≥ 0.5 was considered a large effect size. P-value of less than 0.05 was considered statistically significant.
